# Neural Mechanisms of Visual Dysfunction in Posterior Cortical Atrophy

**DOI:** 10.3389/fneur.2019.00670

**Published:** 2019-06-25

**Authors:** Yi Chen, Ping Liu, Yunyun Wang, Guoping Peng

**Affiliations:** ^1^Department of Neurology, First Affiliated Hospital, Zhejiang University School of Medicine, Hangzhou, China; ^2^Department of Neurology, Shengzhou People's Hospital, Shengzhou, China

**Keywords:** posterior cortical atrophy, visual impairment, visual network, neural mechanisms, neuroimaging

## Abstract

Posterior cortical atrophy (PCA) is characterized predominantly by visual dysfunction that arises from bilateral impairments in occipital, parietal, and temporal regions of the brain. PCA is clinically identified based primarily on visual symptoms and neuroimaging findings. Region-specific gray and white matter deficits have been discussed in detail, and are associated with clinical manifestations that present with similar patterns of perfusion and metabolic findings. Here, we discuss both structural and functional changes in the ventral and dorsal visual streams along with their underlying relationships. We also discuss the most recent developments in neuroimaging characteristics and summarize correlations between distinct neuroimaging presentations.

## Introduction

**Figure 1:**
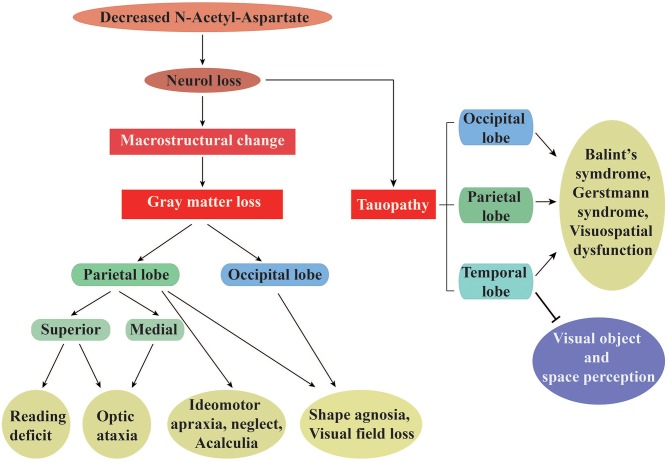
The specific visual symptoms caused by regional changes in gray matter and tauopathy in PCA. (**Left**) A list of the unique symptoms correlated with gray matter loss in parietal or occipital cortices, as well as those correlated with loss in both regions simultaneously. (**Right**) A list of dysfunctions correlated with the temporal, parietal, and occipital lobe tauopathy. Taken together, changes in both gray matter and tau protein are intimately related to neuronal integrity, as assessed by the N-Acetyl-Aspartate levels.

Posterior cortical atrophy (PCA) was initially described by Benson et al. as a progressive neurodegenerative dementia with visual, literary, and numerical deficits (1–4). Patients suffer visuoperceptual, visuospatial, and visuomotor impairments, as well as mathematical, writing, and reading disabilities. Visual field deficits and neuropsychiatric symptoms are also commonly noted, while speaking, memory, and insight are typically preserved in early stages of the condition (3, 4). PCA is a rare condition that typically manifests before 65 years of age and is infrequently first reported in patients over this age (2). As for the pathology of PCA, Alzheimer's disease (AD) accounts for at least 80% of the pathological changes, with the remainder due to the corticobasal degeneration, dementia with Lewy bodies, subcortical gliosis, and prion disease (1, 5). Although AD pharmacotherapy shows promise for PCA management, no study to date has evaluated the efficacy of acetylcholinesterase inhibitors or memantine in patients with PCA (6). PCA mostly arises from AD-like neuropathology and has even been proposed to be a visual variant of AD (7). For instance, biomarker studies have reported that levels of amyloid-β (Aβ), total-tau, and phosphorylated-tau proteins in cerebrospinal fluid and serum profiles of patients with PCA were almost identical to those of patients with AD (5, 8). Other changes in cortical structure and function in parietal, occipital, and temporal regions have been shown to be correlated with clinical and neuropsychological features specific to PCA (see Figure 1). PCA differs from typical AD in that unlike amnesic AD in which the disorder is mainly located in the default mode network, the anatomical and functional damage in PCA is primarily located in higher visual networks (9). Disproportionately asymmetric changes in the posterior cerebral regions is a characteristic of PCA that has been shown using a variety of neuroimaging techniques, including single-photon emission computed tomography perfusion (10), voxel-based morphometry (VBM) for gray matter (GM) (11), diffusion tensor magnetic resonance imaging (DTI) tractography for white matter (WM) (12), positron emission tomography with 18F-labeled fluorodeoxyglucose ([^18^F]FDG-PET) for metabolic evaluation (13), fluorine 18-labeled AV-1451 ([^18^F]AV-1451) PET for pathologic tau (14), and PET with ^11^C-labeled Pittsburgh compound B ([^11^C]PIB) for amyloid deposition (13). Such studies have infinitely improved our understanding of visual dysfunction associated with neurodegenerative conditions.

This review aims to retrospectively summarize the symptoms and mechanisms of visual dysfunction in PCA. Here, we describe changes in cortical functional, the underlying pathological mechanisms, and links to clinically manifesting neuropsychological features. We also summarize neuroimaging characteristics and correlations with distinct clinical presentations.

## Clinical Manifestation

Clinically, PCA manifests with progressive deficits in visuospatial and visuoperceptual abilities and praxis, while memory loss is less prominent (12, 15). PCA patients are characterized by visual agnosia, elements of Balint's (simultanagnosia, optic ataxia, and oculomotor apraxia) and Gerstmann's (acalculia, finger agnosia, left-right disorientation, and agraphia) syndrome, as well as prosopagnosia, topographical disorientation, and limb apraxia (12, 16, 17). Although simultanagnosia has been described as the most common feature of early PCA, the proportion of people who exhibit it varies across studies (18). Deficits in making saccades, fixation stability, and smooth pursuit tasks are also present, and reflected in oculomotor, visuospatial, and visuoperceptual dysfunction (7). Additionally hemianopia has recently been demonstrated to be a visual deficit typical to PCA (19). Reading deficits are also a characteristic of PCA, with reading accuracy adversely influenced by letter size, length, spacing, font, and numbers (20). McMonagle et al. profiled patients who exhibited ventral (i.e., pure alexia, visual agnosia, and prosopagnosia) or dorsal (i.e., ideomotor apraxia, acalculia, simultanagnosia, optic ataxia, and visuospatial neglect) deficits and proposed that PCA can be classified into ventral or dorsal phenotypes (21). Visuospatial language-based learning disabilities are common in PCA, despite preservation of language ability (22). Furthermore, it was unclear what cognitive processes were involved in spelling impairments, and further studies evaluating the impact of peripheral and visuospatial components on spelling deficits are required (23).

## PCA Based on Posterior Cerebral Changes

### Gray Matter

Patients suffering from PCA were found to possess widespread reductions in GM volume, as assessed by VBM. GM atrophy predominantly occurs in the occipital, posterior parietal, and temporal lobes, and is accompanied by cortical thinning in these regions (24). This leads to many of the visuoperceptual and visuospatial deficits mentioned above, as well as less peripheral vision with lateralization (see Figure 1). For example, whole-brain VBM analysis found that decreased reading accuracy, especially during orientation discrimination tasks or visual attention shifting toward peripheral vision, was associated with GM volume loss in the right superior parietal cortex (20). Similarly, optic ataxia in PCA results from a pattern of clustered atrophy in dorsal (superior) and medial parietal regions, while hemifield neglect is typically reported after damage to posterior networks of the temporoparietal and inferior parietal lobes (10). Left-predominant severe ideomotor apraxia is another symptom that has been reported to correlate with GM atrophy in parietal cortex (10). Additionally, loss of visual fields has been shown to be associated with significant reduction of GM in the parietal cortex and lateral and anterior occipital cortices. The greatest visual field loss is contralateral to the side of GM loss, and is typically maximal in the extrastriate regions (19). Experimental findings related to GM volume suggest that focal loss of GM accounts for some of the peculiar clinical features of PCA. For example, spatial attention and shape discrimination scores have been found to correlate with cortical GM volume in the parietal and occipital cortices, while calculation ability has been correlated with GM volume in the parietal cortex (25). GM changes in the lingual, angular, parahippocampal, precuneal, and fusiform gyri, as well as the thalamus and calcarine cortex, warrant further research in PCA (10, 17). Specifically, if patients with PCA manifest with dominant ventral symptoms, GM loss could be localized primarily to bilateral ventral regions (i.e., ventral occipital and temporal cortices). In contrast, dorsal clinical manifestations affecting higher-level processing could be accompanied with greater GM loss in the right inferior parietal, bilateral inferior parietal, and ventral regions (17). Evaluation of GM volume coupled with an appreciation of anatomical cerebral structure is thus a useful approach to localize brain damage.

### White Matter

**Figure 2:**
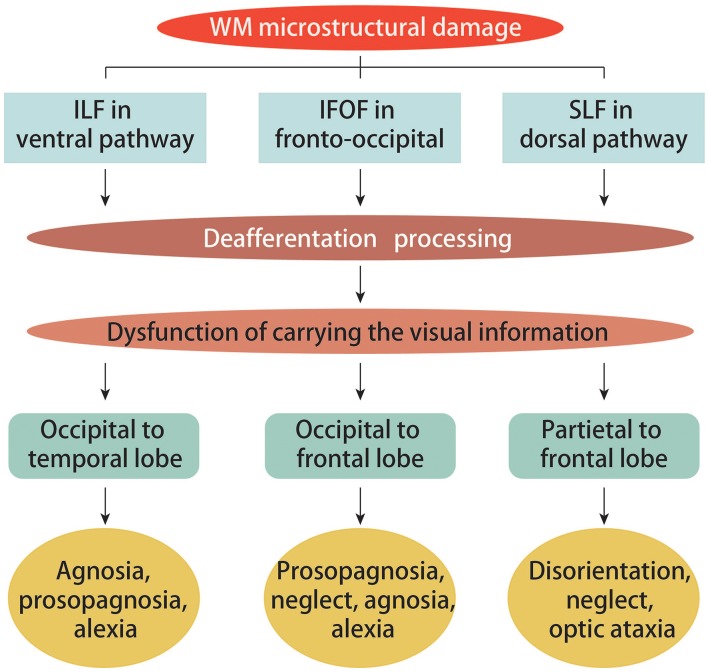
Ventral, fronto-occipital and dorsal structural visual pathways in PCA. White matter (WM) damage across PCA groups was found in the ventral (ILF), fronto-occipital (IFOF), and dorsal (SLF) structural visual pathways. The extensive patterns of damage to the ILF, IFOF, and SLF were most likely a consequence of deafferentation, which has been reported to be crucial for carrying visual information from occipital to temporal regions, occipital to frontal regions, and parietal to frontal regions, respectively. These are the exact factors that contribute to the ventral symptoms (agnosia, prosopagnosia, and alexia), the fronto-occipital pathway symptoms (prosopagnosia, neglect, agnosia, and alexia), as well as the dorsal symptoms (disorientation, neglect, and optic ataxia).

Evaluating alterations of WM in PCA can uncover the underlying neuropsychological and neuroanatomical mechanisms responsible for the disease's pathogenesis. In PCA, diffuse WM damage is typically seen throughout the occipital and temporal ventral regions, dorsal parieto-temporal and ventral occipito-parietal areas, medial structures, and corpus callosum (12, 26). This type of damage is associated with several PCA symptoms. For instance, secondary to posterior cerebral neuronal degeneration, WM atrophy in the callosal splenium was reported as the chief cause for limb apraxia and visual neglect (26). Additionally, as an underlying factor and mediator of simultanagnosia, slower visual processing was found to be associated with WM atrophy in the superior parietal cortex (18). Furthermore, lateralization of degeneration in the visual pathways was marked by damage contralateral to the side most severely affected by visual field loss in the occipital lobe, and progressed into the optic radiation, consistent with GM loss (19). Typically, in patients with PCA, WM microstructural damage is found in the ventral (inferior longitudinal fasciculus, ILF), dorsal (superior longitudinal fasciculus, SLF) and fronto-occipital (inferior fronto-occipital fasciculus, IFOF) visual pathways (see Figure 2), as evidenced by changes in fractional anisotropy (FA) and mean diffusivity (MD) that can be detected via DTI (17, 27). The ILF, SLF, and IFOF have been reported to play crucial roles in conveying visual information as they respectively link occipital areas to the temporal cortex, parietal cortex to frontal cortex, and occipital areas to the frontal cortex (28). The left ILF has also been reported to play a significant role in connecting visual inputs to semantic connotation and subsequent output of correct object recognition; damage to this structure manifests in prosopagnosia, visual agnosia, and alexia. In contrast, right IFOF damage contributes to prosopagnosia and left visual neglect. Impaired SLF tracts result in optic ataxia, visual neglect, and deficits in object localization, especially with greater dorsal involvement (17, 28). Such findings underscore how crucial WM connectivity is to proper cortical function. WM damage follows ventral and dorsal visual processing streams and ultimately affects spatial and object processing (26). Importantly, changes in SLF microstructure have been suggested to correlate with metabolic changes within the inferior parietal and frontal eye field regions (27).

### Functional Correlations Within Cortical Networks

The structural damage associated with PCA appears along with network-level dysfunction, especially in the visual network (9). Recent literature has reviewed ventral and dorsal cortical functionality, including lower and higher components within the cortical hierarchy (29). Cortical activity is meanwhile consistent with that noted in cases of lesions affecting behavior, as confirmed by functional magnetic resonance imaging (fMRI). Behaviorally, patients with PCA exhibit deficits in behavioral visuoconstructive tasks (processing of complex pictures and compound stimuli) in the dorsal visual stream. Likewise, fMRI has revealed that activation within the ventral visual regions is related to facial and object recognition, while that in dorsal areas is related to motion and gestalt perception (29, 30). Visual word processing is a function of the ventral visual stream, and patients with PCA have been reported to exhibit functional deficits in word processing that are seen in reading disorders (31). Although weakened cortical peripheral visual field functions are similar to peripheral visual deficits seen in behavioral pathology, disruption of the dorsal stream has been suggested to result from impaired interconnectivity of feed-forward and -backward pathways.

Using resting-state MRI, functional connectivity (FC) analysis was conducted to assess the specific impairments of PCA (32). As a variant of AD, lower than normal FC was commonly found in the default mode network (DMN) (33). Particularly, a bilateral decrease of FC was observed in the ventral network (occipital and temporal areas) accompanied by severe occipital change. Impairment of the dorsal stream inferior components is ultimately responsible for visual neglect, and even contributes to right-sided neglect (34). Additionally, FC changes in the left inferior parietal cortex has been reported to be related to visual construction and location discrimination abilities (35). Functional hypoconnectivity within the dorsal network has been proposed to mirror compensatory mechanisms early in the course of PCA. Such changes later progress to ventral area hypoconnectivity and likely indicate a persistently progressive pathology (9, 28, 36). One study has shown that further WM damage and FC changes form as the disease duration increased and the severity was greater (28). Thus, more longitudinal studies are needed if we want to understand how the disease evolves or figure out whether combined DTI and functional MRI is a helpful way to monitor its progression.

### Regional Alterations in Perfusion and Metabolism

A study of PCA shows that severe hypoperfusion initially found in the parieto-occipito-temporal cortex subsequently spread to the middle frontal gyri, middle and posterior cingulum, pulvinar, and the postcentral region, bilaterally and symmetrically (8). Strong associations were noted between visual ability profiles and posterior regional hypoperfusion. Aurélie Kas et al., concluded that Gerstmann components (apart from digital agnosia) were linked to perfusion in the left parietal lobe while Balint's components were linked to functions of the superior occipital cortex, superior parietal lobe, precuneus, cuneus, and precentral cortex (8). In patients suffering Gerstmann syndrome, right–left confusion was noted along with dysfunction of the middle temporal cortex, while limb apraxia was associated with left posterior parietal cortical dysfunction. In patients suffering Balint's syndrome, ocular apraxia and ataxia were mainly linked to hypoperfusion of the superior parietal, occipital, and right precuneal regions (the dorsal pathway). Simultanagnosia primarily manifested as a result of higher-level lateral occipital dysfunction (8, 14). Shifts in linear bisection were related to parieto-frontal cortical dysfunction and omissions in target cancellation tests resulted primarily from prefrontal structural damage. Neglect manifested in a mirror-image pattern primarily in the parietal cortex and abnormal eye movements were related to frontal area dysfunction (8, 37, 38). Furthermore, patients with PCA who has a shorter disease course exhibited greater hypoperfusion in posterior cerebral areas (8).

When examined with FDG-PET, patients with PCA showed marked hypometabolism in the posterior regions, with the greatest amount in the higher visual network. These patients had syndromes that were mostly specific to temporooccipital, temporoparietal, and occipito-parietal dysfunction, which were more severe in the right hemisphere than the left (39–41). In addition, symmetrical areas of dysfunction were noted bilaterally in the frontal eye fields, which is responsible for ocular apraxia seen in Balint's syndrome as well as the generation of normal voluntary eye movements under physiologic conditions (42). Hemispheric asymmetry is supposed to arise as an early feature in the course of PCA. One study reported a maximum reduction of metabolism in the occipito-parietal junction (42). Regional cerebral glucose metabolism within the parietal and occipital lobes was identified as associated with optic ataxia (left predominant), simultanagnosia (right predominant), abnormalities in spatial attention and shape discrimination, oculomotor apraxia (extending to the posterior cingulate gyrus), and finally form recognition and object representation that results from temporooccipital dysfunction (25, 39, 40). Interestingly, the severity of regional lesions caused by hypoperfusion and hypometabolism was correlated to the degree of either neuronal loss or synaptic dysfunction, shedding light on advanced visuospatial processing functions (41). Although these findings have been confirmed by cross-sectional design, further verification by longitudinal studies is required.

## Specific Pathologic Features

### Tauopathy

Clinical phenotypes and evolution of AD are closely associated with tau density and spatial distribution of hyperphosphorylated tau [as measured by [^18^F]AV-1451], which are correlated with the degree of neurodegeneration and syndrome localization (14). In cases of higher-order visual processing dysfunctions, AV-1451-PET revealed increased tau accumulation in occipital, parietal, and occipito-temporal regions (see Figure 1). For example, patients with PCA who suffer from Balint's syndrome presented with pathologic findings typical of tau accumulation mostly in lateral and medial occipital, parietal, and temporal cortices, with an asymmetric distribution of pathologic tau in the right hemisphere, but less in the postcentral gyrus, putamen, calcarine fissure, claustrum, hippocampus, thalamus, and subthalamus (14). Deficits in visual object and spatial perception have been confirmed to correlate with greater tau presence in the bilateral occipital lobe, occasionally extending into right temporoparietal areas (43). In addition, elevated neuronal uptake of AV-1451 in cases of PCA revealed cortical atrophy with right lateralization and paralleled hypometabolism (41). The strong negative correlation between GM volume and AV-1451 uptake has suggested that tau pathology could lead to visuospatial and cognitive dysfunctions (which are mediated by GM) (44). Moreover, changes in tau levels have been reported to appear before metabolic dysfunction, suggesting that cerebral metabolism is susceptible to neurotoxicity resulting from tau aggregation (43). How correlations between tau distribution and brain metabolism differ across posterior regions remain unclear. Whitwell et al. systematically summarized the relationships among tau, beta-amyloid, metabolism, and atrophy atypical AD, finding that the strongest correlation was observed between tau-PET and FDG-PET (45). While clinical diagnosis was a variable in this study (45), the correlations between these elements in PCA might differ depending on the imaging method. These relationships therefore need to be verified.

### Amyloid Analysis

Biomarker studies have reported that protein concentrations in cerebrospinal fluid profiles of total-tau, phosphorylated-tau, amyloid-β42 (Aβ42), and the Aβ42/Aβ40 ratio were not useful in differentiating most cases of PCA from typical AD (5, 25). However, [^11^C]PiB PET scans in patients with PCA appear promising (18). Compared with typical AD, patients with PCA exhibited higher PiB uptake in the right posterior lingual gyrus, left middle temporal cortex, inferior frontal cortex, and bilateral occipital lobes with right hemisphere dominance (13, 46). These areas of increased PiB uptake suggest a direct relationship with higher visual networks. PiB network templates suggest a considerable overlap of FDG metabolism in these regions (13, 44). Tau aggregation was found to surpass the number of amyloid deposits, precisely localizing syndromes and clarifying the degree of regional neurodegeneration. Moreover, PIB is less regionally specific than the ^11^C-PBR28 binding pattern, which is a marker for microglial activation-translocator protein (TSPO), primarily in the parietal, occipital, and lateral temporal regions (6, 47). Locations of increased ^11^C-PBR28 binding have been found to overlap with reduced cortical volume or FDG metabolism (46). The limitations of using TSPO-PET in cases of PCA include the limited amount of research and low binding efficiency.

## Conclusion

PCA is a heterogeneous disorder associated with a selective and progressive decline in visual-processing skills. Its pathology has been primarily localized to occipital, parietal, and temporal regions. The mechanisms underlying the visual deficits in PCA are complex and involve multiple cerebral networks. GM and WM structural degeneration are responsible for visual impairments due to the eventual dysfunction of ventral and dorsal processing streams. Changes in functional connectivity account for aberrant communication and neural damage throughout the posterior brain areas, while advanced processing ability within the dorsal stream manifests as visual field deficits. Experiments show that alterations in brain metabolism and perfusion patterns have a reciprocal relationship in visual processing. Tau pathology is more related to the overlap between damaged neuronal regions and their structural and functional correlates. Although our understanding of the macrostructural, microstructural, and neural alterations that give rise to PCA has greatly advanced, the heterogeneity and rarity of this disorder has hindered the development of effective management strategies.

The limitations of the current studies on PCA include: lack of longitudinal studies; the diagnosis criteria was mainly based on clinical symptoms and structural neuroimaging changes; the relationship between different neuroimaging modalities was unclear; and the number of participants was always small in most studies. Therefore, there are series of perspectives in further studies on PCA: firstly, combing DTI and functional MRI to monitor the structural and functional changes during PCA progression with a large number of participants; secondly, using a systematical comparison to measure variability of neuroimaging correlations and underlying mechanisms; thirdly, carrying out higher binding-efficiency TSPO-PET to explore the underlying pathogenesis and effective treatments based on the pathological changes should be recommended.

## Author Contributions

GP and YC: conceived and designed the project. YC and PL: wrote the manuscript with inputs from other authors. YW and YC: drafted the pictures. All authors reviewed and edited the manuscript and approved the final version of the manuscript.

### Conflict of Interest Statement

The authors declare that the research was conducted in the absence of any commercial or financial relationships that could be construed as a potential conflict of interest.
